# Engineering CuZnOAl_2_O_3_ Catalyst for Enhancing CO_2_ Hydrogenation to Methanol

**DOI:** 10.3390/molecules30061350

**Published:** 2025-03-18

**Authors:** Peixiang Shi, Jiahao Han, Yuhao Tian, Jingjing Wang, Yongkang Lv, Yanchun Li, Xinghua Zhang, Congming Li

**Affiliations:** 1 State Key Laboratory of Clean and Efficient Coal Utilization, College of Chemistry and Chemical Engineering, Taiyuan University of Technology, Taiyuan 030024, Chinaliyanchun01@tyut.edu.cn (Y.L.); 2 College of Safety and Emergency Management, Taiyuan University of Technology, Taiyuan 030024, China

**Keywords:** CO_2_, methanol, CuZnOAl_2_O_3_, pretreatment, carbonate

## Abstract

The CuZnOAl_2_O_3_ catalyst shows excellent activity and selectivity in the reaction of CO_2_ hydrogenation to methanol as a consequence of its controllable physicochemical properties, which is expected to offer an efficient route to renewable energy. In this study, CuZnOAl_2_O_3_ catalysts are engineered by a special pretreatment, constructing a carbonate structure on the surface of the catalyst. Compared to the unmodified catalyst, the optimized catalyst (CZA-H-C1) not only exhibits an improved methanol selectivity of 62.5% (250 °C and 3 MPa) but also retains a minimal degree of deactivation of 9.57% over a 100 h period. By characterizing the catalysts with XRD, TEM, XPS and in situ DRIFTS spectroscopy, it was found that the surface carbonate species on Cu-based catalysts could significantly enhance the reaction and shield the active sites. This study offers theoretical insights and practical strategies for the rational design and optimization of high-performance heterogeneous catalysts.

## 1. Introduction

The combustion of fossil fuels gives rise to a sharp rise in CO_2_ emissions, causing a serious greenhouse effect [1,2]. Notwithstanding CO_2_ is a small molecule, it can be used as a valuable resource for sustainable development [3,4,5]. Methanol, one of the products derived from CO_2_ conversion, serves as a crucial chemical feedstock and a clean energy source [6]. Upon a sustainable chemical synthesis, CO_2_ combining with green hydrogen to produce methanol shows a great potential to reduce carbon emissions, achieving fuel appreciation and economic efficiency [7]. To develop highly active catalysts, CuZnOAl_2_O_3_ is considered as one of promising candidates in CO_2_-to-methanol conversion.

CO_2_ hydrogenation to methanol normally accompanies the formation of CO originating from a reverse water–gas shift (RWGS). The overall process is relatively complex, and the energy conversion efficiency of this reaction is largely dependent on thermodynamics. Considering the chemical inertness of CO_2_, temperatures employed in the reaction typically exceed 240 °C [8]. However, the continuous high temperature and competition of side reactions lead to the low selectivity and stability of CuZnOAl_2_O_3_ catalysts. Liang et al. conducted an online test on CuZnOAl_2_O_3_ catalyst for 720 h, and the long-term test resulted in a 34.5% reduction in the space–time yield of CH_3_OH [9]. In addition, other Cu-based catalysis showed a kinetic complexity and some degree of limitation in thermodynamics [10,11]. Typically, the activation of CO_2_ and the co-adsorption of H_2_ took place on the CuZnOAl_2_O_3_ catalyst simultaneously. The co-adsorbed hydrogen could induce the dissociation of CO_2_ and convert it into CO, surface (O*) and surface hydroxyl (HO*), which were subsequently converted into carbonate (CO_3_*), bicarbonate (HCO_3_*) and formate (HCOO*) [12]. It was reported that the bonding strength of these intermediates is related to the engineered structure of the catalysts [13]. Zhou et al. designed a silica-supported Cu/Mo_2_CT_x_ catalyst interface for CO_2_ hydrogenation to methanol and identified that these types of interfaces incur steady intermediates of HCOO* and CH_3_O* [14]. Although designing a catalyst interface has proven to be effective in the hydrogenation of CO_2_, there is still a great challenge for practical applications.

According to previous studies, catalysts can be modified via improving the preparation method, adding auxiliaries and wrapping the active site, and such approaches enhance the favorable characteristics of catalysts. For example, the introduction of a strong metal–support interaction (SMSI) into a Cu-based catalyst through a single solid precursor-derived preparation method can improve the dispersion and sintering resistance of the catalyst, thereby improving its long-term stability [15]. Moreover, the inclusion of additives can change the electronic properties and surface active sites of the catalyst, thus improving the activity and selectivity of the catalyst. Zhang et al. designed the Cu^+^/CeZrO_x_ interface by adding Ce and Zr to regulate the electronic state of Cu and stabilize the *CO intermediate [16]. Apart from catalyst modifications, the pretreatment of the catalyst is also one of the key steps in catalyst engineering. Cu-based catalysts are usually pretreated by traditional H_2_ reduction, which has a single effect and low efficiency [17]. Due to the strong reducing ability of H_2_, most CuO is usually reduced to Cu^0^, resulting in the same high activity to the RWGS reaction and the reduction in methanol selectivity. In our earlier studies, although the valence state of Cu was adjusted by CO reduction, it failed to obtain a long-term stable effect [18]. Zhu et al. found that the addition of a certain proportion of H_2_O/CH_3_OH mixture in the hydrogen reduction of commercial CuZnOAl_2_O_3_ catalysts can induce the migration of ZnO species from the support to the surface of metal copper particles [19]. This highlights the importance of a multi-dimensional pretreatment in tuning the surface properties of catalysts.

To address inadequate energy conversion, the surface structure of CuZnOAl_2_O_3_ catalyst was engineered with a special pretreatment that facilitated the formation of surface carbonate species. This pretreatment enhanced the dynamic equilibrium of the reaction, thereby promoting CO_2_ hydrogenation to methanol. Subsequently, the physicochemical properties of the catalyst were characterized. The results showed that the proper CO_2_ pretreatment could generate a certain amount of carbonates on the catalyst surface. This not only promotes reaction balance and methanol selectivity but also protects the active sites and enhances the catalyst’s stability.

## 2. Results and Discussion

### 2.1. Crystal Structural and Morphological Particle Size of the Catalyst

**Figure 1:**
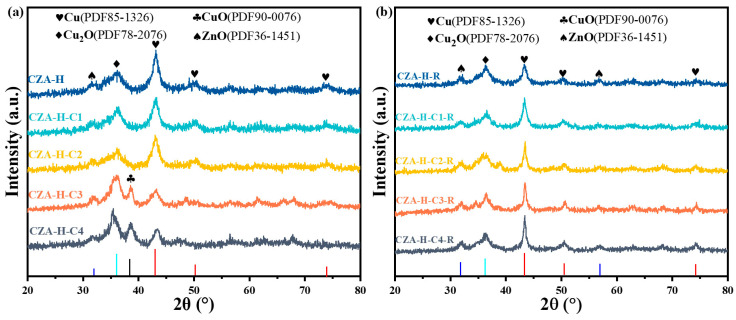
XRD patterns of CZA catalyst after pretreatment (**a**) and after reaction (**b**).

The crystal structure of the catalysts after pretreatment and reaction were characterized by XRD, as shown in Figure 1. Figure 1a compares the structure of catalysts pretreated in different manners. The CZA-H catalyst presented the characteristic Cu_2_O (PDF78-2076) diffraction peak at 36.3°and Cu (PDF85-1326) diffraction peak at 43.3°. With an increasing CO_2_ pretreatment time, the peak intensity of Cu diffraction decreased, while the half-peak width increased. CuO (PDF90-0076) diffraction peaks at 35.5° and 38.6° were detected in the CZA-H-C3 and CZA-H-C4 catalysts. These results indicated that the valence state and crystal size of copper species changed during the pretreatment process. The diffraction peaks of Cu_2_O and Cu were mainly detected in the catalyst after reaction, as shown in Figure 1b. It is to be noted that the crystal structures were similar. This finding showed that the oxidation and reduction rates were different during the reaction. It indicated that increasing the pretreatment time not only led to a change in valence state of Cu species from a highly reduced state to a highly oxidized state, but it also resulted in the variation of the particle size of Cu species. Due to the high reaction temperature, Cu species might be sintered and grow, eventually leading to an alteration in catalyst structure [20]. According to Scherrer’s formula, the particle size of Cu species can be calculated from the half-peak width of the diffraction peak. The grain size of Cu species after pretreatment is between 7 and 13 nm, while the average grain size of Cu species after reaction is about 20 nm, which is in line with the corresponding TEM images. In addition, only weak ZnO peaks and no Al peaks were detected in the XRD pattern. Therefore, we confirmed their content and ratio by EDS mapping imaging (Figure S2 and Table S1).

**Figure 2:**
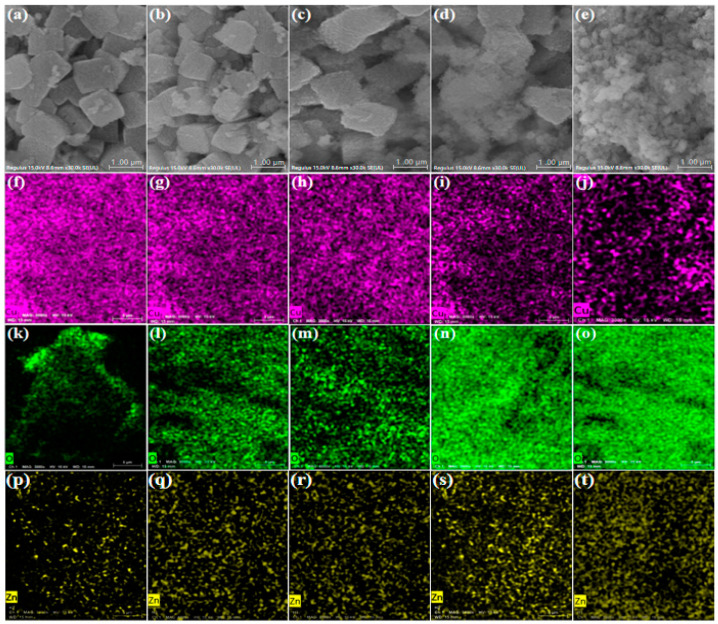
SEM images of the activated CZA catalyst (**a**–**e**) CZA-H, CZA-H-C1, CZA-H-C2, CZA-H-C3, CZA-H-C4 and elemental energy spectrum: (**f**–**j**) Cu; (**k**–**o**) O; (**p**–**t**) Zn. (legend: MAG: 3000×, HV: 15 kV, WD: 15 mm).

The morphology and distribution of elements of catalysts after pretreatment were studied by SEM equipped with EDS mapping as shown in Figure 2. In Figure 2a–e, the regular cubic block species was formed by the reduction of H_2_ on the CZA-H catalyst, which had a larger pore gap. A low-power HD image of the CZA-H-1 catalyst is shown in Figure S3 for reference. After the CO_2_ pretreatment, the massive accumulation structure of cubic block species in the CZA-H-C1 catalyst began to collapse. Increasing the CO_2_ pretreatment time resulted in the occurrence of aggregation, until agglomeration spherical particles were formed in the CZA-H-C4 catalyst. According to the previous report, Cu species are expected to be a major composition in these cuboidal particles [21]. To visualize the changes of composition in each species, Cu, O and Zn were characterized by EDS mapping. With the pretreatment of CO_2_, the Cu element is gradually dispersed, while the distribution of the O element increased with time due to the presence of copper oxides. The distribution of Zn is almost unaffected during the pretreatment process. The C element is unable to be accurately identified by SEM for the analysis of carbonate species. This will be further demonstrated by the TEM characterization.

In order to prove whether the structural collapse of the catalyst occurred during the pretreatment process, the specific surface area and pore volume of the catalyst were characterized by N_2_ adsorption–desorption. As shown in Table 1, the specific surface area of the catalyst increased and then decreased during the pretreatment process. The increase in the surface area could be the consequence of the formation of carbonates on the catalyst surface, and the following decrease is probably related to the oxidation state transformation of copper species and high-temperature agglomeration, which eventually caused the collapse of the pore structure [22]. The pore volume variation of the catalyst was consistent with the above results.

**Figure 3:**
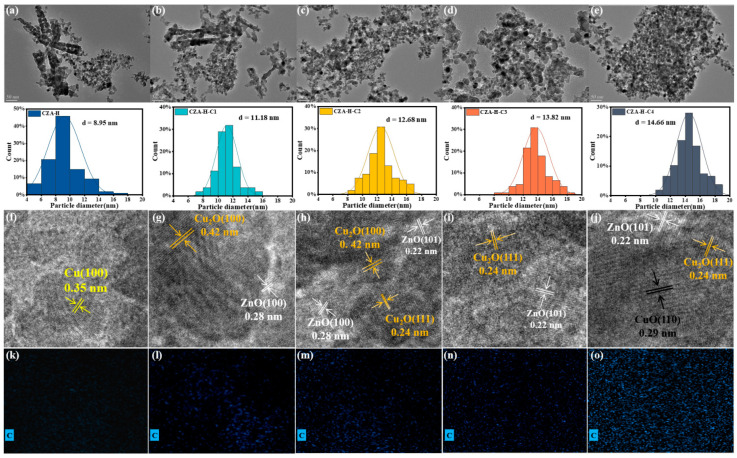
TEM image of activated CZA catalyst: (**a**–**e**) low multiple (50 nm); (**f**–**j**) high multiple (5 nm). Statistical distribution of particle size and energy spectrum of elements: C (**k**–**o**).

The particle size and energy spectrum analysis of the C element were characterized by TEM, as shown in Figure 3. The particles started with both cubic and granular forms (Figure 3a,b), and with the increase in the CO_2_ pretreatment time, all the cube crystals disappeared and changed into the granular form. According to a particle size statistical histogram, the relevant particle size increases with a longer pretreatment time (from 8.95 nm to 14.66 nm). Table 2 compared the particle size of Cu species measured with different characterization techniques, among which the particle size obtained by TEM was regarded as the most accurate. The species distribution of the catalyst under different pretreatment times was obtained by calculating the lattice fringes of high-resolution electron microscopy [23]. The Cu (100) crystal surface detected on the CZA-H catalyst was reduced by the traditional H_2_ reduction. With prolonging the pretreatment time, Cu (100) was gradually converted into Cu_2_O (100) and Cu_2_O (111) under the action of CO_2_, which was consistent with the XRD results indicating the partial oxidation of Cu species. However, carbonate species cannot be directly detected. The energy spectrum of the carbon element indicated the distribution of the C element was gradually more dense with the increase in CO_2_ pretreatment time. Combined with the O element energy spectrum analysis, the formation of carbonate species could be determined. The pretreated catalyst was characterized by H_2_-TPR to assess its secondary reduction ability. Figure S1 shows that the reduction peak gradually shifted to high temperatures with the increase in CO_2_ pretreatment time, and the H_2_ consumption increased. According to the previous report, this also implied the formation of carbonate [24].

### 2.2. Electronic State of Elements on the Catalyst Surface

**Figure 4:**
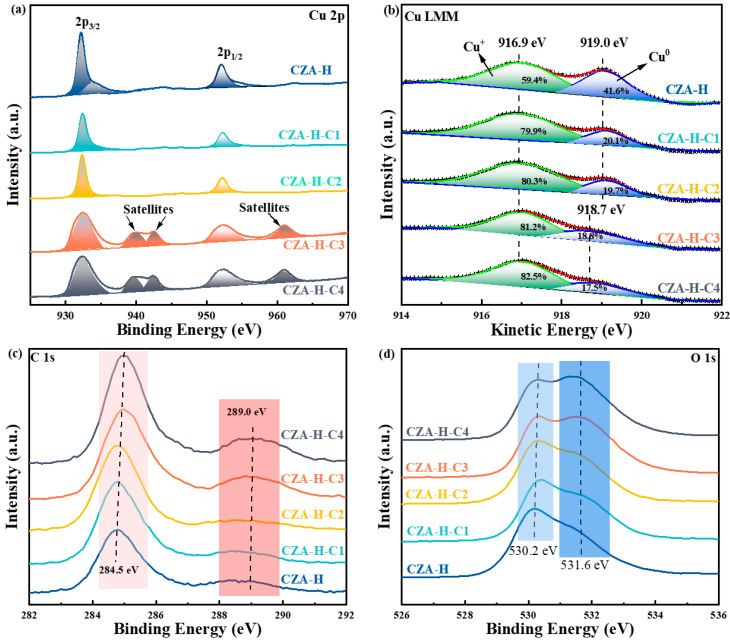
XPS results of CZA catalysts after different pretreatments: (**a**) Cu 2p; (**b**) Cu LMM; (**c**) C 1s; (**d**) O 1s.

XPS was used to study the chemical states of Cu species on the surface of the CZA catalysts and determine the formation of carbonate species as shown in Figure 4. Figure 4a shows the Cu 2p spectrum of the pretreated CZA catalyst. The CZA-H catalyst primarily displayed two distinct BE peaks, at 932.2 eV and 952.1 eV, corresponding to Cu 2p_3/2_ and Cu 2p_1/2_. Upon increasing the CO_2_ pretreatment time, the CZA-H-C1 and CZA-H-C2 catalysts showed weaker Cu 2p_3/2_ and Cu 2p_1/2_ peaks compared with the CZA-H catalyst, indicating that the amount of Cu^0^ or Cu^+^ decreased gradually [25]. Due to the small chemical shift of Cu to Cu_2_O, it was difficult to distinguish them by photoelectron spectral lines alone [26]. The appearance of satellite peaks and the weak shift of Cu 2p_3/2_ peaks towards a high binding energy in the CZA-H-C3 and CZA-H-C4 catalysts manifested an increase in copper oxidation [27].

The Cu LMM XAES spectra showed a gradual decrease in the content of Cu^0^. It is worth noting that the Cu^0^ peak position of the catalysts of CZA-H-C3 and CZA-H-C4 were shifted due to the electronic interaction between a certain substance and the Cu species. The results of the deconvolution based on Cu LMM spectra are shown in Table S2. According to the tabulated data, after H_2_ reduction and CO_2_ pretreatment at different times, the Cu LMM XAES deconvolution results showed that the Cu^+^ content gradually increased from 59.4% to 82.5%, while the Cu^0^ content gradually decreased from 41.6% to 17.5%. This resulted in a significant increase in the Cu^+^/Cu^0^ ratio from 1.43 to 4.71. This could be related to excessive carbonate, which was confirmed by the C 1S and O 1s spectra of the pretreated catalyst (Figure 4c,d) [28]. Prior to CO_2_ pretreatment, the standard peak of the C element at 284.5 eV represented the presence of amorphous carbon and normally served as a standard material. There was a wide small crest in the range of 288 to 290 eV, reflecting the presence of partial hydroxyl carbon, which was derived from partial oxides or hydrolysates on the catalyst surface [29]. As the CO_2_ pretreatment time increased, the main peak position of C 1s gradually shifted towards the direction of higher binding energy, and the carbonate peak appeared at 289.5 eV [30]. As for the O 1s spectrum, the peak of metal oxide at 530.2 eV was assigned to the combination of oxygen and metal. The gradual shift of the peak at 531.6 eV towards a higher binding energy demonstrated the presence of carbon oxide on the catalyst surface, which can be explained by the generation of carbonate species [31].

### 2.3. Analysis of the Adsorption Ability of the Cu-Based Catalyst

**Figure 5:**
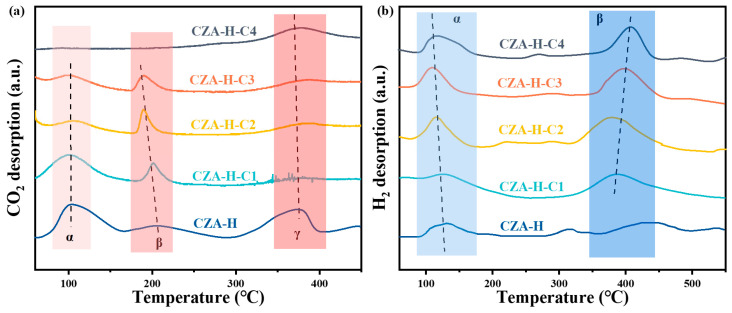
Temperature programmed desorption curve of pretreated catalysts: (**a**) CO_2_-TPD and (**b**) H_2_-TPD.

To investigate the catalyst surface and adsorption characteristics, the TPD patterns of CO_2_ and H_2_ for the pretreated CZA catalysts are depicted in Figure 5, alongside the data of the CZA catalyst subjected to a CO_2_-free pretreatment. According to the CO_2_-TPD, each specimen exhibited three main peak profiles: a small α peak at approximately 100 °C indicative of weak desorption, a moderate β peak at around 200 °C representing moderate desorption and a pronounced γ peak at approximately 370 °C signifying strong desorption [32]. Changes in the peak areas of the samples suggested varying intensities of CO_2_ adsorption on the pretreated CZA catalysts. The low-temperature peak was associated with surface Brønsted basic sites, such as -OH groups, whereas the medium-temperature peak was related to metal–oxygen pairs [33,34]. The intensities of the α and γ peaks decreased with an increase in the CO_2_ pretreatment time, indicating that the number of alkaline sites available for adsorption on the catalyst surface decreased. Moreover, the adsorption sites were no longer active due to pore collapse, surface area reduction or chemical reaction or physical blockage [35]. For the moderate alkaline adsorption peak, the peak area slightly changed, but the peak location shifted to the low temperature, indicating that the alkali strength of the adsorption site declined. As to the strong basic interaction, CO_2_ adsorption substantially influenced the catalytic efficiency for CO_2_ hydrogenation to methanol [36]. The CO_2_-TPD desorption peak of the pretreated catalyst was deconvolution calculated, and the results are shown in Table S3. With the increase in CO_2_ pretreatment time, the amount of CO_2_ desorption in the low-temperature zone gradually decreased, the amount of CO_2_ desorption in the middle-temperature zone gradually increased, and the amount of CO_2_ desorption in the high-temperature zone gradually decreased. In addition, the formation of carbonate was expected to occupy a certain CO_2_ adsorption site, which also proved the existence of carbonate. H_2_, being an additional reactant, also exhibits two main desorption peaks in Figure 5b, with the α peak and the β peak at approximately 110 °C and 380 °C, respectively. The α desorption peak at lower temperatures was ascribed to either the physical adsorption of H_2_ or chemical adsorption at less intense sites, such as surface defects [37]. With the increase in CO_2_ treatment time, the area of the α peak of the catalyst gradually increased. The high-temperature desorption β peaks corresponded to the strong H_2_ adsorption at more stable adsorption sites (reduced Cu^0^ or metal–carrier interactions that might be formed at metal oxide interfaces) [38]. The increase in peak area suggested that the number of sites available for hydrogen adsorption on the catalyst increased, or the adsorption strength of the adsorption site increased. The shift in peak position to high temperature implied that more carbonate compounds were formed on the catalyst surface, which was more favorable for H_2_ adsorption. According to the spectrogram, it was found that the CZA-H-C1 and CZA-H-C2 catalysts were relatively superior to others in the adsorption and activation of reactants.

### 2.4. In Situ DRIFTS Analysis of Catalyst Surface Dynamics

**Figure 6:**
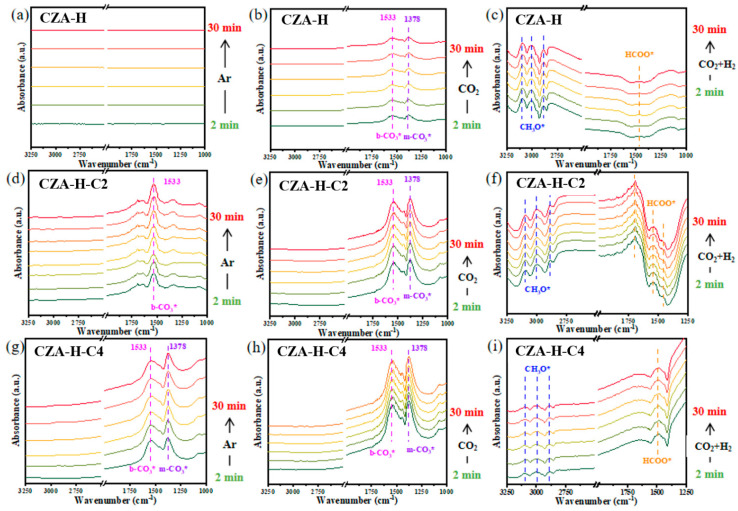
In situ DRIFT spectra of CZA catalysts after different pretreatments: (**a–c**) CZA-H; (**d–f**) CZA-H-C2; (**g–i**) CZA-H-C4 exposed to Ar, CO_2_ and CO_2_/H_2_ atmospheres, respectively.

Feedstock gas adsorption was examined via in situ DRIFT spectroscopy to identify surface reaction intermediates and confirm the significance of carbonates in the hydrogenation of CO_2_, as shown in Figure 6. Initially, the catalyst was purged with Ar for 30 min. Notably, no absorption bands were observed in the spectrum of the reduced CZA-H catalyst. However, the exposure of the CZA-H-C2 catalyst to an Ar atmosphere showed a bidentate carbonate (b-CO_3_*) peak near 1533 cm^−1^. Meanwhile, the peak of monodentate carbonate (m-CO_3_*) was also observed at 1378 cm^−1^ on the CZA-H-C4 catalyst. This indicated the development of carbonate species during the catalyst’s surface post-pretreatment and the superiority of b-CO_3_* over m-CO_3_* [39,40]. The main reason for this difference is that the three catalysts have undergone different degrees of CO_2_ pretreatment, which leads to the difference in carbonate formation on the catalyst surface. When the atmosphere was switched to CO_2_, peaks of both carbonate species were detected in all cases. Compared with the previous peak intensity, the peak intensity increased in the order of CZA-H-C4 > CZA-H-C2 > CZA-H. Despite the presence of carbonates on the pretreated catalyst surface, there were still CO_2_ adsorption activation sites. According to the characterization of the CO_2_-TPD, surface carbonate species occupied part of the CO_2_ adsorption and activation sites, which were mainly dominated by bidentate carbonate, and the formation of this species was related to the adsorption of strong alkaline [41,42]. Finally, the catalysts were exposed to the reaction gas (CO_2_/H_2_) atmosphere. In Figure 6c,f,i, the peaks of formate (HCOO*) species and methoxyl (CH_3_O*) species are mainly detected in the atmospheric reaction state, serving as the main intermediates of methanol synthesis reaction. As to IR spectra, the absorption bands at about 3000 cm^−1^ were assigned to the asymmetric and symmetric stretching vibrations of the C-H bond in CH_3_O* [43,44]. There was a band near 2888 cm^−1^, indicating the C-H stretching mode of CH_3_O* species on Cu sites. There were intense adsorption bands at 1533 cm^−1^ and 1378 cm^−1^ corresponding to the asymmetric and symmetric O-C-O stretching modes, which were indicative of formate (HCOO*) and carbonate (CO_3_*) intermediates adsorbed on the surface. It has been verified that formate species (HCOO*) are crucial intermediates in the methanol synthesis process over Cu-based catalysts [45,46]. A stronger HCOO* peak was detected in the CZA-H-C2 catalyst, indicating that proper CO_2_ pretreatment was more conducive to the conversion of reactants on the catalyst surface [47]. According to the in situ DRIFT characterization results, CO_2_ hydrogenation to methanol with the traditional H_2_ reduction state of the Cu-based catalyst involved several steps: CO_2_ activation, H_2_ dissociation, the formation of the HCOO* intermediate, hydrogenation, dehydroxylation to CH_3_O* and the production of methanol. The difference in the catalysts in this study is that the Cu-based catalyst surface will be induced to form carbonate species after CO_2_ pretreatment, which can directly react with the dissociated H*. It reduces the steps of activating CO_2_ in the initial stage of the reaction and empowers a faster reaction speed.

### 2.5. Catalyst Performance

**Figure 7:**
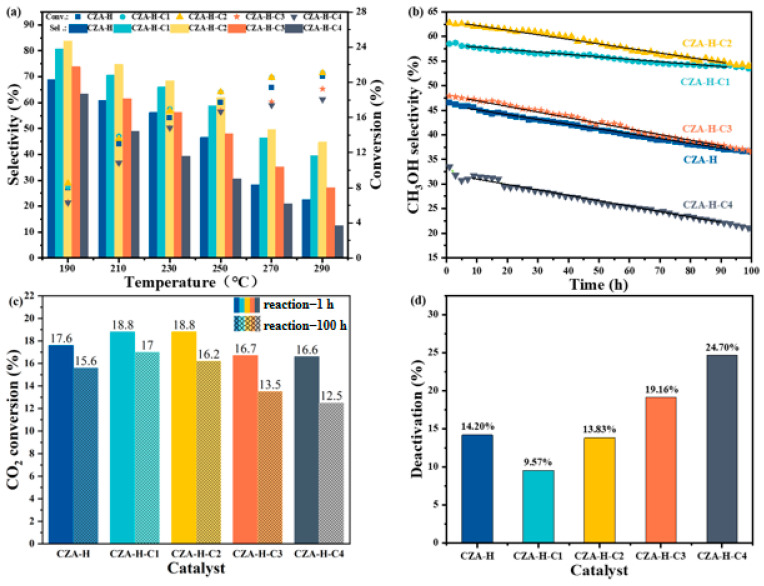
Performance assessment results of CZA catalyst after activation at different CO_2_ times: (**a**) CO_2_ conversion and CH_3_OH selectivity (reaction conditions: 190–290 °C, 3.0 MPa); (**b**) CH_3_OH selectivity change during reaction; (**c**) comparison of CO_2_ conversion before and after 100 h of reaction; (**d**) deactivation degree after 100 h of reaction (reaction conditions: 250 °C, 3.0 MPa).

The evaluation of the pretreated catalyst used for CO_2_ hydrogenation to methanol is shown in Figure 7. Figure 7a presents the change in CO_2_ conversion and CH_3_OH selectivity at 190–290 °C and 3 MPa. The CO_2_ conversion of all the catalysts increased with the increase in temperature and gradually became stable after reaching 250 °C, confirming that CO_2_ was normally completely activated above 240 °C. The CO_2_ conversion increased from 6% at 190 °C to 20% at 290 °C in this process. Under the same conditions, the CO_2_ conversion of the CZA-H-C1 and CZA-H-C2 catalysts was higher, reaching to about 18.8% at 250 °C. However, the CO_2_ conversion of the CZA-H-C3 and CZA-H-C4 catalysts was about 16.6%, suggesting that the increase in carbonate species might block the CO_2_ activation sites. In addition, the methanol selectivity of all the samples decreased gradually with the increase in reaction temperature, but the methanol selectivity decreased with varied rates with CZA catalysts using different pretreatments. Moreover, with the change in CO_2_ pretreatment time, the methanol selectivity of different CZA catalysts varied at the same temperature. For example, the methanol selectivity of CZA-H-C2 can reach as high as 62.5% at 250 °C, which was about 15% higher than that of the traditional H_2_ pretreatment CZA-H catalyst, whereas that of the CZA-H-C4 catalyst was only 31% at 250 °C. This change aligns with the variation in formate peak intensity. Therefore, it can be concluded that surface carbonates are likely involved in the reaction, transforming into formate species and eventually enhancing the reaction rate.

The catalyst was then subjected to a 100 h stability test, and the results are shown in Figure 7b. The comparison revealed that the methanol selectivity for all the catalysts declined with an extended reaction time, suggesting that the catalysts were not adequately stable over such long periods of reaction. However, the methanol selectivity of the different pretreated catalysts exhibited different decreasing trends. The overall rate of decline was CZA-H-C4 > CZA-H-C3 > CZA-H > CZA-H-C2 > CZA-H-C1. Figure 7c also shows the change in CO_2_ conversion for 1 h and 100 h. By calculating the deactivation degree of each catalyst after a 100 h reaction, it was found that the deactivation degree of the CZA-H catalyst reduced by hydrogen was 14.20%, while the deactivation degree of the CZA-H-C1 catalyst was the lowest, only 9.57%. It indicated that the appropriate amount of carbonate species on the catalyst surface can not only improve methanol selectivity but also protect the active sites and improve catalyst stability. However, an excess of carbonates was detrimental to the catalyst’s activity and stability. Thus, the appropriate CO_2_ pretreatment of Cu-based catalysts could enhance both catalytic performance and stability. Table 3 compares the performance of Cu-based catalysts for CO_2_ hydrogenation to methanol from recent studies. It is noteworthy that the CZA-H-C2 catalyst in this study outperforms the majority of previously reported Cu-based catalysts for this reaction. The reason why the pretreatment catalyst we studied has a higher catalytic activity is that compared with the H_2_ reduction activation of the traditional Cu-based catalyst, the CO_2_ pretreatment step is added, which covers the surface of the CZA catalyst with a layer of a carbonate structure. The carbonate structure layer can not only quickly induce the formation of methanol intermediates during the reaction. Increasing the selectivity of CH_3_OH can also protect the active site to avoid the oxidation and deactivation of surface-active Cu species, thus improving the stability of the catalyst. Therefore, the CZA-H-C2 catalyst developed by us has certain advantages over the reported Cu-based catalysts.

### 2.6. Effect of Carbonate

**Figure 8:**
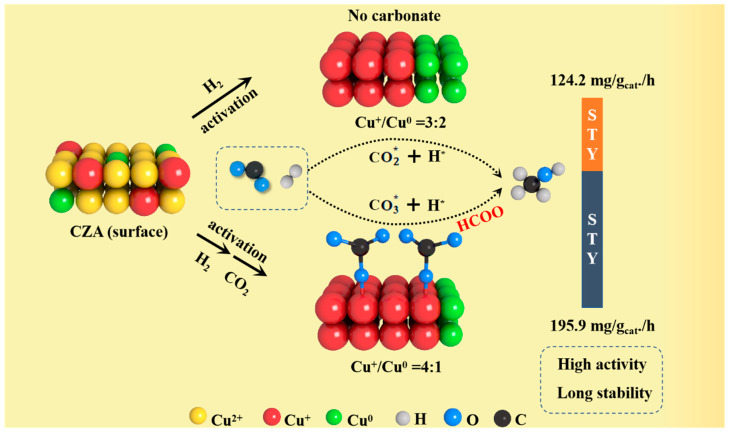
The key role of carbonate in reaction homeostasis.

Compared with H_2_ reduction, the difference with the CO_2_ pretreatment of Cu-based catalysts is that H_2_ reduction usually involves the use of hydrogen as a reducing agent, while CO_2_ pretreatment only uses CO_2_ in the reaction process to improve the catalytic performance, confirmed by the structural and electronic state changes [57]. According to the above results, the carbonate species were formed on the surface of the CZA catalyst by H_2_ reduction and proper CO_2_ pretreatment. Combined with the characterization and evaluation results, the carbonate species played a pivotal role in CO_2_ hydrogenation to methanol. Firstly, as one of the reaction intermediates, carbonate can induce the reaction into dynamic equilibrium more quickly. The presence of carbonate promoted the adsorption and dissociation of H_2_, which accelerated the next transition to the intermediate formate [58]. The reaction equilibrium K constants at 250 °C and 3 MPa are listed in Table 4. The standard equilibrium constant of CO_2_ hydrogenation to methanol under this condition was 1.55 × 10^−3^. With the increase in carbonate species on the catalyst surface, the reaction equilibrium constant was closer to the standard value. According to the results of the experiment and characterization analysis, the core content of this work is converted into graphic form, as shown in Figure 8. It can be seen that a Cu-based catalyst modified by surface carbonate was more favorable to the CO_2_ hydrogenation to methanol reaction. In addition, carbonate species formed on the surface of the Cu-based catalyst can also be used as a physical structural agent to protect the active sites and avoid agglomeration and oxidation. Carbonate can form a stable coating layer on the catalyst surface, which can effectively reduce the direct contact between Cu particles and inhibit the agglomeration of Cu [59]. Additionally, the carbonate coating protected Cu species from oxidizing agents. In an oxidizing environment, the carbonate layer can prevent the contact of oxygen with Cu species, thus reducing the oxidation of Cu. This can be proved by the calculation of the deactivation degree after 100 h tests. The deactivation degree of the CZA-H-C1 catalyst was 4.63%, which was inferior to the CZA-H catalyst.

## 3. Experimental Section

### 3.1. Catalyst Preparation

The CuZnOAl_2_O_3_ catalyst was prepared by the coprecipitation method. Firstly, 7.490 g copper nitrate trihydrate, 4.613 g zinc nitrate hexahydrate and 0.971 g aluminum nitrate nonahydrate were dissolved in deionized water to form a 0.5 M salt solution. Meanwhile, 10.6 g of anhydrous sodium carbonate was dissolved in a certain amount of deionized water to form 0.5 M of alkaline solution. After being fully dissolved, the alkali solution was slowly injected into the salt solution and stirred in the water bath at 68 °C. The pH of the mixture was stabilized at 7.0 ± 0.2 by adjusting the injection rate. The suspension was gradually formed, and the mixture was further aged for 1 h after the titration completed. The precipitates were filtrated and washed with 3000 mL of deionized water, followed by drying at 80 °C overnight and calcining at 350 °C for 4 h. The as-prepared catalyst was denoted as CZA.

### 3.2. Catalyst Pretreatment

The CZA catalyst was pretreated via traditional H_2_ reduction and H_2_ plus CO_2_ reduction–oxidation. Firstly, the CZA catalyst was reduced by 10% H_2_/N_2_ at 60 mL/min for 1 h, which was marked as CZA-H. After that, 10% CO_2_/N_2_ at 60 mL/min continued to be injected for different times ranging from 1 to 4 h. The obtained catalysts were denoted as CZA-H-C1, CZA-H-C2, CZA-H-C3 and CZA-H-C4, respectively. The aforementioned pretreatment was conducted at a temperature of 300 °C, and the resultant catalysts were subsequently stored under vacuum conditions.

### 3.3. Catalyst Characterization

XRD patterns was collected using a TD-M001 X-ray diffractometer (Bruker, Bremen, Germany) (at 40 kV and 30 mA using Cu Kα radiation, 2θ range 20° to 80°) equipped with a graphite filter. The average dimensions of the Cu-based nanoparticles were determined by applying the Scherrer equation.

(1) $$d_{Cu}=\frac{k\lambda }{\beta cos\theta }$$

The above parameters represent the full width at half-maximum (β), diffraction angle (θ), Scherrer constant (k = 0.89) and X-ray wavelength (λ = 0.154 nm), respectively.

The N_2_ adsorption and desorption were performed using a 3H-2000PS2 apparatus (BeiSiDe, Beijing, China). Herein, the specific surface area and microporous surface area were determined using the Brunauer–Emmett–Teller (BET) (BeiSiDe, Beijing, China) theory and the T-plot approach, respectively, and the mean pore diameter and volume were calculated by the Barrett–Joyner–Halenda (BJH) model.

The dispersion and morphology of Cu species were observed by SEM on the Regulus 8100 (Hitachi, Tokyo, Japan), and the distribution of elements was detected by energy-dispersive spectroscopy (EDS). A 40 mg sample was loaded on the sample tray and charged under the accelerating voltage.

TEM was conducted using JEOJEM-2100 (JEOL Ltd., Tokyo, Japan). The catalysts were dispersed into ethanol with the assistance of ultrasonication. A few drops of suspension were placed on a carbon-coated copper net. The measurement was operated at 200 kV.

H_2_-TPR analysis was conducted using the FINESORB-3010 apparatus (Jingwei Gaobo Science and Technology Co., Ltd., Beijing, China). An amount of 40 mg of the sample was placed into a U-shaped quartz reactor. The reaction tube was heated to 250 °C at a rate of 10 °C/min, and 30 mL/min Ar was injected to remove the surface impurities of the catalyst, and finally the 30 mL/min 10 vol% H_2_/Ar was heated to 600 °C at a rate of 10 °C/min for reduction.

A Thermo Fisher Scientific ESCALAB 250Xi instrument (Thermo Fisher Scientific, Waltham, MA, USA) was used for XPS analysis. The energy spectrum was collected under Al Kα radiation (*h*_υ_ = 1486.6 eV). Prior to the measurements, binding energy (BE) values were calibrated against 284.6 eV of the C 1s peak, assuring the accuracy of the data. The positions of photoelectron peaks indicated their binding energy (BE), and Auger peaks represented their kinetic energy (KE).

CO_2_-TPD was performed on the Micromeritics AutoChem 2920 (Micromeritics Instrument Corporation, Norcross, GA, USA). Catalysts (0.06 g) in a U-tube were pretreated under Ar at 50 °C with a flow rate of 30 mL/min, then saturated with pure CO_2_ for 1 h. After an Ar purge for 30 min, the catalysts were heated to 800 °C at 10 °C/min. The measurement of H_2_-TPD was the same as with CO_2_-TPD, except replacing pure CO_2_ with 10 vol% H_2_/Ar.

The in situ DRIFTS was analyzed on a Nicolet 6700 (Thermo Fisher, Waltham, MA, USA). Catalysts were pretreated at 200 °C in a pure Ar atmosphere for 30 min. They were then exposed to 10% CO_2_/Ar at 250 °C for 30 min, followed by a switch to a 3:1 H_2_/CO_2_ mixture for an additional 30 min. The spectra were deconvoluted to estimate species quantities by integrating peak areas after background subtraction and cycle tests.

### 3.4. Catalytic Testing

A fixed-bed reactor was used to evaluate the performance of the CZA catalysts. A 50 mg sample of 20–40 mesh catalyst was placed into a reaction tube, which was packed with matching quartz sand above and below the catalyst. A reaction gas mixture of H_2_ (72%), CO_2_ (24%) and Ar (4%) was injected into the reactor at a rate of 30 mL/min. The pressure was rapidly increased to 3 MPa, followed by heating the reactor between 190 and 290 °C to study the temperature’s effect. CO and CO_2_ concentrations were determined using a GC with a TCD and two columns (a 5A molecular sieve and a GDX-104 packed column (Panna, Changzhou, China)). Methanol was detected by an FID using a Porapak-Q column (Panna, Changzhou, China). Ar was introduced in the feedstock as a reference. The CO_2_ conversion rates and selectivity of the products for each catalyst were calculated using the following equations:

(2) $$X_{{CO}_{2}}(\%)=\left(1-\frac{A_{out(CO_{2})}/A_{out(Ar)}}{A_{in(CO_{2})}/A_{in(Ar)}}\right)\times 100\%$$

where $A_{in(CO_{2})}$ and $A_{in(Ar)}$ were the pre-reaction peak areas of CO_2_ and Ar, and $A_{out(CO_{2})}$ and $A_{out(Ar)}$ were the post-reaction peak areas of CO_2_ and Ar.

(3) $$S(CO)(\%)=\frac{\frac{A_{out\left(CO\right)}}{A_{out\left(Ar\right)}}\times f_{\frac{CO}{Ar}}}{\left(\frac{A_{in\left(CO_{2}\right)}}{A_{in\left(Ar\right)}}-\frac{A_{out\left(CO_{2}\right)}}{A_{out\left(Ar\right)}}\right)\times f_{\frac{CO_{2}}{Ar}}}\times 100\%$$

(4) $$S\left(CH_{3}OH\right)\left(\%\right)=\left(\frac{A_{CH_{3}OH}\times f_{CH_{3}OH}}{\left(\frac{A_{in\left(CO_{2}\right)}}{A_{in\left(Ar\right)}}-\frac{A_{out\left(CO_{2}\right)}}{A_{out\left(Ar\right)}}\right)\times f_{\frac{CO_{2}}{Ar}}}\right)\times 100\%$$

where $A_{out(CO)}$ and $A_{CH_{3}OH}$ were the peak areas of CO and CH_3_OH, respectively. $f_{CO/Ar}$, $f_{CO_{2}/Ar}$ and $f_{CH_{3}OH}$, respectively, correspond to the correction factors of CO, CO_2_ and CH_3_OH.

## 4. Conclusions

To sum up, the effects of surface carbonate modification on a CuZnOAl_2_O_3_ catalyst for CO_2_ to methanol has been studied. The surface properties of the CZA catalysts (producing carbonate species) are changed by adjusting the CO_2_ pretreatment time based on H_2_ reduction, which is more tunable compared with the traditional single H_2_ reduction. The relevant characterization results show that the presence of carbonate species on the catalyst surface is conducive to the stabilization of the valence state of Cu species, while promoting the adsorption and dissociation of H_2_, which can promote the formation of formate and accelerate the hydrogenation of CO_2_ to methanol. Compared with the CZA-H catalyst (46.6%), the engineered CZA-H-C2 (62.5%) catalyst exhibited a higher CH_3_OH selectivity at 250 °C and 3 MPa. Meanwhile, the stability of the CZA-H-C1 catalyst was remarkably improved (the deactivation degree decreased by 4.63% compared to CZA-H) for 100 h, which was attributed to the protection of the carbonate formed on the surface. These results provide significant insights for the development of highly effective catalysts and the refinement of CO_2_ hydrogenation processes.

## Figures and Tables

**Table 1:** N_2_ adsorption–desorption results of CZA catalysts with different activation treatments.

Sample	S_BET_ (m^2^/g_cat._)	V_total_ (cm^3^/g)	D_BJH_ (nm)
CZA-H	36.51	0.61	48.48
CZA-H-C1	43.12	1.06	65.87
CZA-H-C2	38.25	0.83	50.24
CZA-H-C3	30.16	0.41	36.07
CZA-H-C4	26.99	0.29	29.67

**Table 2:** The particle size of pretreated CZA catalysts.

Sample	d_Cu_/nm (XRD)	d_Cu_/nm (SEM)	d_Cu_/nm (TEM)
CZA-H	7.52	9.80	8.95
CZA-H-C1	8.90	11.59	11.18
CZA-H-C2	10.05	13.21	12.68
CZA-H-C3	11.42	14.63	13.82
CZA-H-C4	12.55	15.87	14.66

**Table 3:** Comparative analysis of catalytic activities among various Cu-based catalysts.

Sample	T/°C	P/MPa	CO_2_ Conv./%	CH_3_OH Sel./%	Ref.
CZA	250	3	19.0	50.0	[48]
CZ-5H_2_	280	3	5.6	62.0	[49]
CZA-CP	250	3	17.5	44	[50]
Cu/ZnO/C-P	250	3	12.7	80.7	[51]
Ce-CuZn-MOF	260	2.8	8.0	71.1	[52]
Si-Cu-Zn	240	3	4.7	29	[15]
Cu/ZnO/Al_2_O_3_	240	4.6	21.0	39.0	[53]
Cu-ZnO-SrTiO_3_	250	3	18.9	38.0	[54]
Cu/ZnO/ZrO_2_	240	3	15.7	58.0	[55]
CZAZ	250	3	14.2	57.2	[56]
CZA-H-C2	250	3	18.9	62.1	This work

**Table 4:** The equilibrium constant K of catalysts under corresponding reaction conditions.

Sample	T/°C	P/MPa	K ^1^	Deact. ^2^/%
Standard	250	3	1.55 × 10^−3^	-
CZA-H	250	3	5.43 × 10^−4^	14.20
CZA-H-C1	250	3	1.08 × 10^−3^	9.57
CZA-H-C2	250	3	1.24 × 10^−3^	13.83

^1^ $K=\frac{n(CH_{3}OH)\times n(H_{2}O)}{n(CO_{2})\times n^{3}(H_{2})}$. ^2^ Deactivation degree of catalyst reaction for 100 h.

## Data Availability

Data are contained within the article and Supplementary Materials.

## Supplementary Materials

The following supporting information can be downloaded at: https://www.mdpi.com/article/10.3390/molecules30061350/s1, Figure S1: H_2_-TPR spectra of CZA catalysts after different pretreatments; Figure S2: EDS-mapping analysis of CZA catalyst after calcination; Figure S3: The SEM image of CZA-R-1 catalyst at low power; Table S1: composition distribution of elements in catalyst; Table S2: Deconvolution results of Cu LMM XAES of activated CZA catalysts; Table S3: CO_2_ desorption capabilities of the CZA catalysts after pretreatment.
