# Supplementary material for: Engineering CuZnOAl2O3 Catalyst for Enhancing CO2 Hydrogenation to Methanol
Source: Molecules. 2025 Mar 18;30(6):1350. doi: 10.3390/molecules30061350 (PMC11946585; doi:10.3390/molecules30061350)
Supplement: Supplementary file 1 [file molecules-30-01350-s001.zip › molecules-3528874-supplementary.pdf]

Supporting information

Engineering $CuZnOAl_2O_3$ catalyst for enhancing CO_2 hydrogenation to methanol

Peixiang Shi[a, b], Jiahao Han[a], Yuhao Tian[a], Jingjing Wang[a], Yongkang Lv[a], Yanchun Li[a] , Xinghua Zhang[b, *], Congming Li[a, *]

[a]: State Key Laboratory of Clean and Efficient Coal Utilization, College of Chemistry and Chemical Engineering, Taiyuan University of Technology, Taiyuan 030024, Shanxi, China.

[b]: College of Safety and Emergency Management, Taiyuan University of Technology, Taiyuan 030024, Shanxi, China.

Corresponding authors: Xinghua Zhang; Congming Li

Email addresses: zxh1969@163.com;

licongming0523@163.com

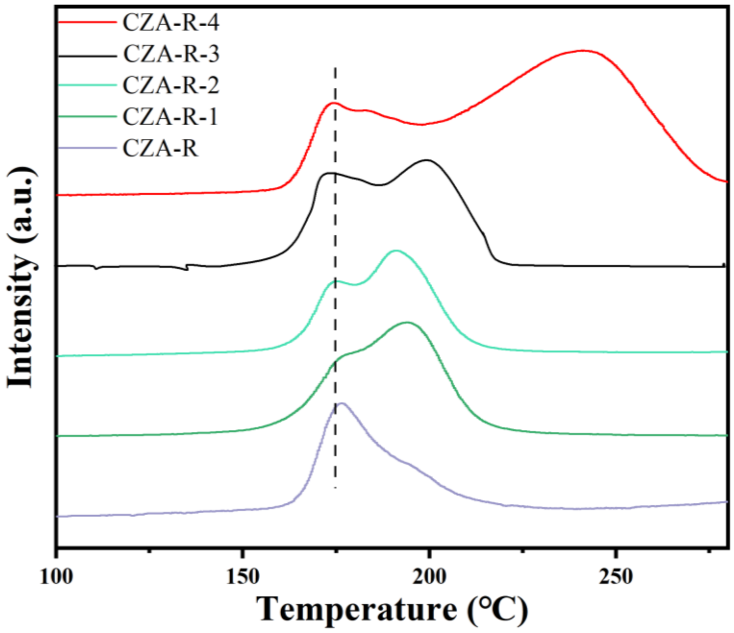

Fig. S1. H_2-TPR spectra of CZA catalysts after different pretreatments.

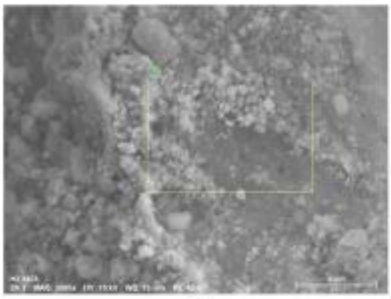

名称	日期	时间	HV [kV]	放大倍数	WD [mm]
H2 1408	1/12/2024	10:09:13 AM	15.0 keV	3000x	15.0 mm

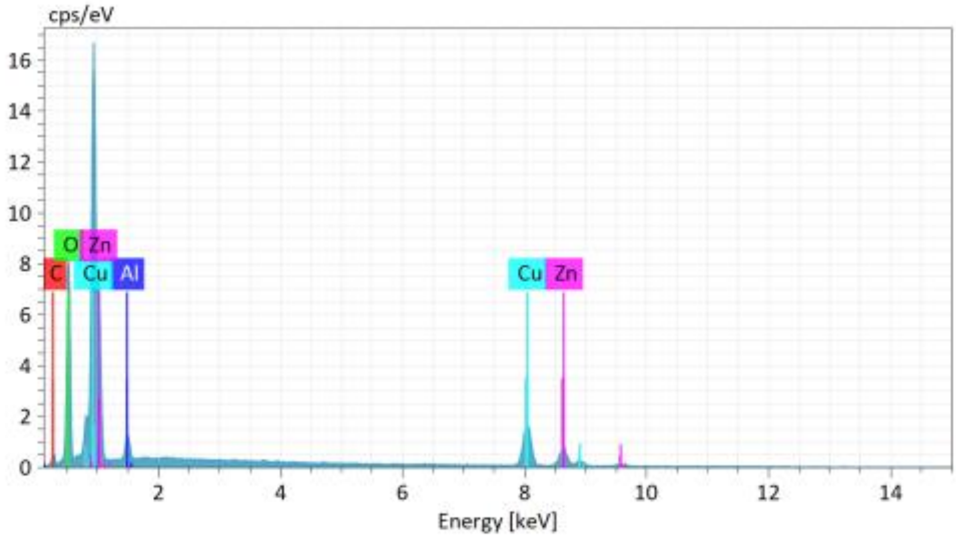

Fig. S2. EDS-mapping analysis of CZA catalyst after calcination.

Table S1. composition distribution of elements in catalyst.

Catalysts	Element	Mass percentage content (wt %)
CZAl	Cu	57.420
	Zn	32.519
	Al	10.061

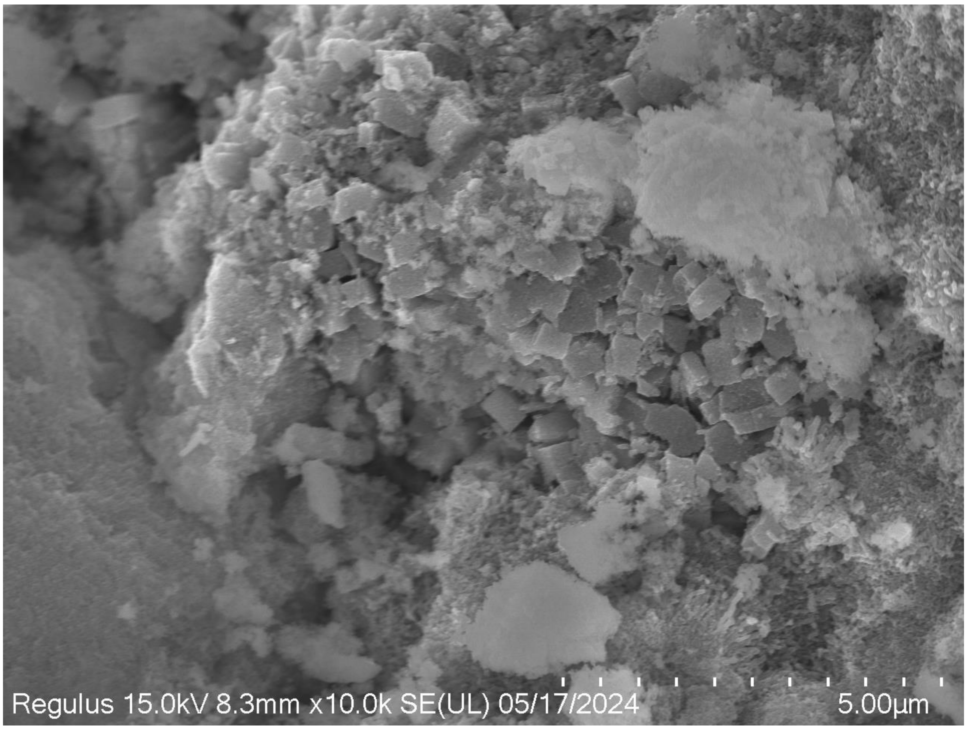

Fig. S3. The SEM image of CZA-R-1 catalyst at low power.

Table S2. Deconvolution results of Cu LMM XAES of activated CZA catalysts.

Catalyst	Cu^+ (%)	Cu^0 (%)	Cu^+/Cu^0
CZA-R	59.4	41.6	1.43
CZA-R-1	79.9	20.1	3.98
CZA-R-2	80.3	19.7	4.08
CZA-R-3	81.2	18.8	4.32
CZA-R-4	82.5	17.5	4.71

Table S3. CO_2 desorption capabilities of the CZA catalysts after pretreatment.

Sample	CO_2 desorption amount (μmol·g^{-1})		
	Low temperature peak	Medium temperature peak	High temperature peak
CZA-R	10.01 (101.5℃)	4.06 (205.1℃)	8.80 (367.2℃)
CZA-R-1	9.23 (101.1℃)	6.16 (200.2℃)	9.06 (368.3℃)
CZA-R-2	3.39 (101.2℃)	6.86 (196.8℃)	6.02 (369.0℃)
CZA-R-3	3.20 (100.5℃)	7.07 (192.7℃)	3.10 (370.2℃)
CZA-R-4	-	-	4.04 (368.1℃)
